# Experimentally Informed Numerical Simulations of Spray Deposition and Runoff Doses in a 10-Day-Old Nose Model

**DOI:** 10.3390/ph19020217

**Published:** 2026-01-27

**Authors:** Jack Yongfeng Zhang, Mary Ziping Luo, Ray Lameng Lei, Sung-An Lin, Xiuhua Si, Jinxiang Xi

**Affiliations:** 1 Amphastar Pharmaceuticals, Inc., Rancho Cucamonga, CA 91730, USA; jyz@amphastar.com (J.Y.Z.); maryl@ims-limited.com (M.Z.L.); rayl@amphastar.com (R.L.L.); andrewl@amphastar.com (S.-A.L.); 2 Department of Aerospace, Industrial, and Mechanical Engineering, California Baptist University, Riverside, CA 92504, USA; asi@calbaptist.edu; 3 Department of Biomedical Engineering, University of Massachusetts, Lowell, MA 01854, USA

**Keywords:** nasal sprays, Eulerian Wall-Film model, physiology-based simulations, infant nose model, runoff, supine position, porcine nasal mucosa, liquid film thickness

## Abstract

**Background:** Intranasal drug delivery is a preferred route for emergency administration of naloxone in opioid overdose due to its rapid onset of action and ease of use. However, limited knowledge exists on the delivery efficiency and safety of nasal sprays in neonates, particularly in life-threatening situations such as coma states where breathing is compromised. This study presents a physiology-based simulation of spray deposition and runoff loss in a 10-day-old infant nose model. **Methods:** Spray characteristics, including droplet size distribution, exiting velocity, and plume angle, were measured and implemented in ANSYS Fluent droplet tracking model. Naloxone film thickness was measured on ex vivo porcine nasal mucosa at varying angles and used in the Eulerian Wall-Film model. Simulations were conducted in a 10-day-old nose geometry across multiple doses (0.25, 0.50, 1.0, and 2.0 mL) in supine and 45° inclined postures to quantify regional deposition, liquid film translocation, and pharyngeal runoff. **Results:** While a 0.25 mL spray was fully retained in the nasal passages, higher doses exceeded the mucosal holding capacity and caused significant runoff. Runoff into the pharynx was 18.5% and 10.1% for the spray volume of 0.50 mL in the 45° back tilt and supine positions, respectively. The 1.0 mL spray caused 55.1% and 53.5% runoff in the 45° back tilt and supine positions, while the 2.0 mL spray caused 77.5% and 76.8% runoff in the 45° back tilt and supine positions, respectively. **Conclusions:** These findings highlight the critical influence of spray volume on drug delivery outcomes in neonates and provide quantitative guidance for optimizing intranasal naloxone administration in emergency pediatric care.

## 1. Introduction

Opioid overdose remains a major public health crisis, with naloxone recognized as the first-line emergency treatment due to its rapid opioid receptor antagonism [1,2,3]. While intranasal (IN) delivery of naloxone has been widely adopted in adults and older children, its application in neonates and young infants remains poorly understood [4,5,6,7]. This knowledge gap is particularly critical because opioid exposure in neonates, whether from maternal transfer, accidental ingestion, or iatrogenic causes, can result in respiratory depression and coma, requiring immediate intervention [8,9,10,11]. In such circumstances, intravenous or intramuscular administration is often impractical, and intranasal delivery becomes the most accessible and life-saving route.

The unique challenges of administering intranasal sprays to neonates arise from their markedly different nasal anatomy compared to older children and adults [12,13,14,15]. Newborn nasal passages are narrower, have smaller surface area, and lower mucosal liquid-holding capacity, all of which significantly alter deposition patterns and increase the likelihood of runoff into the pharynx, which may further pose a greater risk of choking to neonates [16,17,18,19]. Moreover, a neonate in a coma state may have severely reduced ventilation or apnea, further compromising drug transport by airflow and altering spray deposition and runoff behavior [20]. These factors raise critical questions about the efficacy, safety, and optimal dosing strategies for IN naloxone in this vulnerable population.

Computational fluid dynamics (CFD) modeling has become an important complement to in vitro and clinical studies for nasal products; several CFD studies have employed wall-film models to quantify deposition efficiency and runoff. It has been demonstrated that spray dose, body position, and nasal surface area strongly influence drug distribution, and that doses exceeding the mucosal holding capacity result in significant runoff into the pharynx [21,22,23,24,25,26,27]. Spray–wall interactions and post-deposition liquid motion (i.e., wall-film formation, gravity-driven translocation, and shear/surface-tension effects) strongly influence the final regional dose distributions after an IN spray [28,29,30,31]. Validation and sensitivity analyses further support CFD as a cost-efficient framework to evaluate device parameters, head orientation, and breathing conditions that are impractical to probe exhaustively in bench or clinical settings [32]. Still, most studies focus on adult or older pediatric anatomies; data and simulations tailored to neonates, especially within the first weeks of life, remain sparse [33].

The objectives of this work are twofold, as follows: (1) to characterize liquid–wall adhesion on ex vivo porcine nasal mucosa and enhance physiological realism of the Eulerian Wall-Film model, and (2) to assess spray deposition and runoff in an infant nasal geometry. By providing insight into the impacts and mechanisms of device-, delivery-, and patient-associated parameters governing intranasal drug delivery in infants, this study seeks to explore optimized dosing strategies for neonatal opioid overdose interventions. Specific aims include the following:

(1) To measure the deposited liquid layer thickness on nasal mucosa freshly extracted from suckling pigs at different inclination angles and implement them into a numerical model.
(2) To experimentally characterize nasal spray properties, including droplet size distribution, exit velocity, and plume angle.
(3) To simulate the initial deposition distribution in a 10-day-old nasal model under varying parameters related to device (plume angle, exit velocity, and droplet size distribution), delivery (insertion depth, nozzle angle, and dose), and patient (body position).
(4) To simulate post-deposition liquid translocation and quantify runoff after applying sprays of 0.25 mL, 0.50 mL, 1.0 mL, and 2.0 mL in 45° back tilt and supine positions.

## 2. Results

### 2.1. Spray Retention on Ex Vivo Porcine Nasal Mucosa at Varying Angles

**Figure 1:**
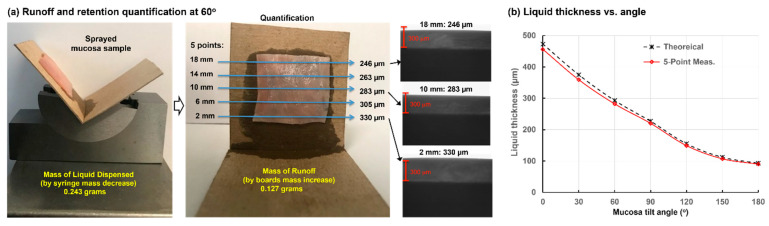
Porcine mucosa runoff and retention testing with 0.25 mL sprayed on a 20 mm × 20 mm mucosa sample: (**a**) mucosa positioned at a 60° tilt angle with runoff quantification and liquid thickness measurements averaged across five points on the sample; and (**b**) theoretical and five-point average measured liquid film thickness as a function of mucosa tilt angle.

Figure 1a shows the spray runoff and retained liquid height on porcine nasal mucosa at a 60° tilt angle. In this case, 0.243 g of naloxone HCL solution was applied onto the mucosa sample measuring 20 mm × 20 mm. The runoff onto the supporting boards can be seen in the rightmost panel of Figure 1a. The second panel in Figure 1a shows the same sample from a different view that better displays the runoff condition. Based on the mass difference of the board(s), it was determined that 0.127 g of the 0.243 g applied ran off this particular sample. Also shown is the positioning of five points (second panel, Figure 1a) where the liquid film thicknesses were measured based on grayscale analyses of the image tangent to the surface. Thickness images from three positions are illustrated in the third panel of Figure 1a, with a 300 µm scale bar (red bar) superimposed onto the liquid layer image for comparison. Thicknesses were found to have a gradient on all tilted samples, with the lowest (downside) portions of the mucosa being thicker.

Table 1 shows ten trials of measurements related to spray runoff and retention on porcine mucosa samples at 60° tilt, including the mass of syringe and cardboard before/after administration and the optical film thicknesses at five points. Table 2 lists the calculated mass of applied sprays and runoff for each trial, as well as the residual mass on the mucosa, the theoretical film thickness assuming uniform distribution, and the 5-point average measurement. These two thicknesses agreed closely, with the measurement value slightly less than the theoretical value (−3.6% ± 1.4%). This trend is reasonable in that any potential runoff, either from splash or evaporation, will underestimate the mucosa residual mass, yielding a lower measured film thickness.

Figure 1b shows the theoretical and five-point-average measured thicknesses as a function of mucosa tilt angle ranging from 0° to 180°. For each angle, ten trials similar to those performed at 60° tilt were conducted, and the data in this figure represents 70 trials in total. As expected, film thickness decreased with increasing angle of nasal mucosa tilt. Good agreement was observed for all tilt angles between the theoretical and measured thicknesses, with theoretical values averaging less than 4% higher than measured values. This indicates that a good recovery—more than 96% of the retained naloxone solution on nasal mucosa was accounted for by optical thickness measurements.

### 2.2. Spray Characterization and Modeling: Size, Velocity, and Plume Angle

The size distribution of the nasal spray droplets was measured using a laser diffraction particle size analyzer (Spraylink, Dickinson, TX, USA). The volume-based distribution Dv(10), Dv(50), and Dv(90) values were 28.1 ± 3.2 µm, 66.0 ± 14.2 µm, and 145.3 ± 29.8 µm, respectively. The spray plume angle was gauged to be around 45°. Short durations of ~0.1 s were segmented and used for particle imaging velocimetry (PIV) analysis to quantify the droplet speeds. Due to the coalescence, breakup, and evaporation of the droplets, it is difficult for PIV to track individual droplets in order to calculate droplet speeds reliably; therefore, only high-quality images within a short duration were used for this purpose. The calculated speed was estimated to be around 8.5 ± 3.5 m/s, which was implemented in the spray model for subsequent numerical simulations.

### 2.3. Parametric Studies of Varying Factors on Initial Deposition

#### 2.3.1. Nozzle Orientation, Exit Velocity, and Droplet Size

**Figure 2:**
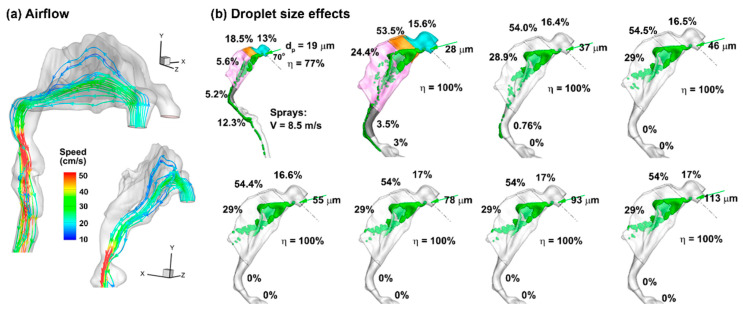
Airflow and inert particle dynamics: (**a**) airflow field in the right nasal passage at an inhalation flow rate of 0.76 L/min, and (**b**) effects of particle size (19–113 µm) on the initial deposition pattern and regional deposition fraction for a given delivery scenario (i.e., a head orientation of 45° back tilt, a nozzle angle of 45° relative to the nostril normal), and a 2 mm insertion depth into the nostril. The cyan color in (**b**) denotes nasal vestibule, the brown color denotes the valve, and the pink color denotes the turbinate region.

The airflow field in the 10-day-old nose model is shown in Figure 2a in terms of streamlines in the right passage at an inhalation flow rate of 0.76 L/min (i.e., 20% of the normal breathing rate, representing a coma state). The inlet Reynolds number is around 214, and the flow is laminar. In this study, the airflow speed is one order of magnitude lower than that of the droplets. Moreover, the density of air is three orders of magnitude lower than that of the liquid. Thus, the transport and deposition of liquid droplets are predominately dictated by inertial impact and not noticeably affected by airflow details.

The effect of the droplet size (i.e., 19–113 µm) on the initial deposition distribution is shown in Figure 2b, with a back-tilted body position of 45° and a nozzle angle of 45° relative to the nostril normal. The deposition distributions are similar when the droplet size is larger than 28 µm, although the regional deposition fractions may vary slightly (less than ±1%, as shown in Figure 2b). The surface deposition patterns of 19 µm droplets are notably distinct from those of larger droplets, which deposit across the whole nasal airway. A small fraction of 28 µm droplets is observed to deposit beyond the nasopharynx (3.5%), which is absent for droplets of 37 µm and larger. However, the deposition pattern of 28 µm droplets in the front nose is already similar to those of 37 µm droplets and larger. This deposition similarity for 37 µm droplets and larger support the feasibility of studying only one representative droplet size to represent all droplets larger than 28 µm. As a result, subsequent simulations consider only the Dv50 size (66 µm).

#### 2.3.2. Effect of Spray Release Position and Orientation

**Figure 3:**
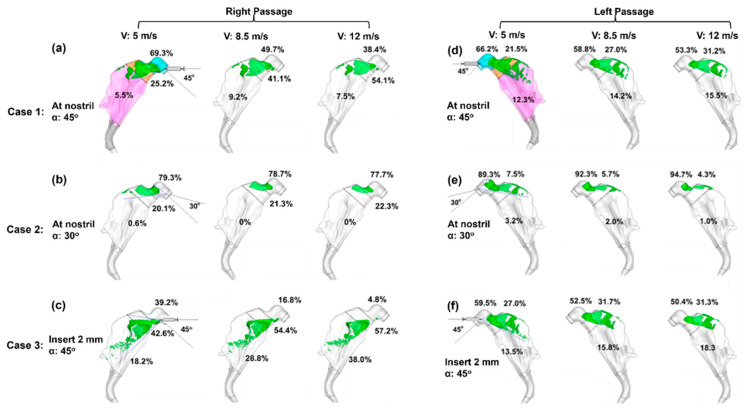
Initial surface deposition for 66 µm aerosols under three delivery scenarios with varying nozzle position and orientation in the right (**a**–**c**) and left (**d**–**f**) passages: (**a**,**d**) at the nostril opening with a nozzle angle of 45° (Case 1), (**b**,**e**) at the nostril opening with a nozzle angle of 30° (Case 2), and (**c**,**f**) with a 2 mm insertion into the nostril at a nozzle angle of 45° (Case 3). The cyan color in (**a**,**d**) denotes nasal vestibule, the brown color denotes the valve, and the pink color denotes the turbinate region.

To consider the effects of spray release position, three delivery protocols with varying nozzle positions and angles were considered (Figure 3). In Case 1, the spray nozzle was positioned at the nostril opening with a 45° angle relative to the nostril normal direction, while in Case 2, with a 30° angle. In Case 3, the nozzle was inserted 2 mm into the vestibule with a 45° angle. For each case, three droplet exit speeds (5 m/s, 8.5 m/s and 12 m/s) were considered for both nasal passages.

Considering the right nasal passage (Figure 3a), large discrepancies are observed among the three cases, indicating a significant impact from the spray release position and orientation. The droplet exit velocity is observed to exert a smaller impact on the deposition pattern, which appears to be velocity-independent when V ≥ 8.5 m/s (Figure 3a–c). However, a quantitative comparison of regional deposition fractions reveals dose shifts with velocity in each case despite their visual resemblance. In Case 3 (Figure 3c), the vestibular deposition fraction (DF) decreases from 39.2% for V = 5 m/s, to 16.8% for V = 8.5 m/s, and to 4.8% for V = 12 m/s; meanwhile, the turbinate DF correspondingly increases from 18.2% to 28.8% to 38.0%. In Case 1, 5.5–9.2% reaches the turbinate’s right passage, (Figure 3a), while in Case 2, almost no sprays reach that region (Figure 3b). Both cases dispense lower doses to the turbinate than Case 3 for both right and left passages.

Different deposition patterns arise between the left and right nasal passages for each delivery scenario, reflecting their different morphologies. Considering the left nasal passage, 12.3–15.5% of sprays deposit in the turbinate region in Case 1 (Figure 3d) and 13.5–18.3% in Case 3 (Figure 3f), while in Case 2, nearly all sprays are filtered out by the vestibule and valve, with a negligible fraction (1.0–3.2%) reaching the turbinate (Figure 3e). In the sections to follow, all test cases adopt the Case 3 delivery protocol (insertion of 2 mm with a nozzle angle of 45° relative to the nostril normal), and a Eulerian Wall-Film (EWF) model is applied to simulate the liquid film translocation and dose distribution.

### 2.4. Liquid Film Translocation

#### 2.4.1. Delivered Dose at a 45° Back Tilt Body/Head Position

**Figure 4:**
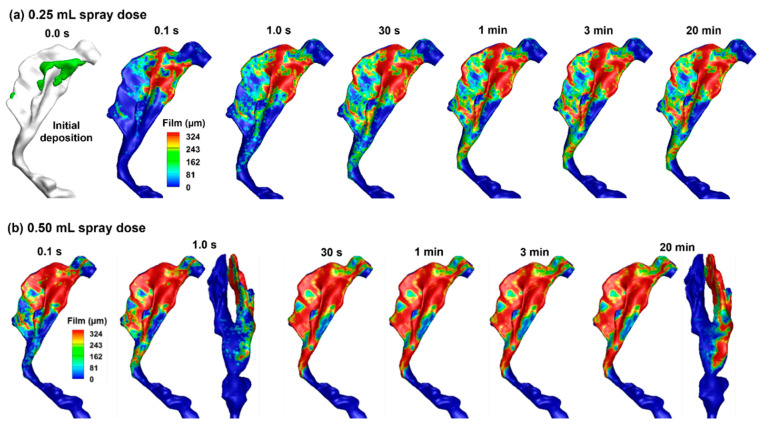
Liquid film translocation vs. time in the right nasal passage with a 45° back tilt position and a spray dose of (**a**) 0.25 mL and (**b**) 0.50 mL.

Once the droplets are deposited on the airway surfaces, a liquid film forms, which may remain stationary or move. For nasal sprays applied to an infant, it is important to determine whether runoff occurs and, if so, how much. Figure 4a shows the thickness of the liquid film at 0.1 s, 1 s, 30 s, 3 min, and 20 min with an applied spray dose of 0.25 mL. The initial droplet deposition (without film spreading) is also plotted in Figure 4a for comparison. It is observed that, when neglecting tissue absorption, the liquid film stabilizes at 1 s and exhibits no significant variation thereafter. With a spray dose of 0.25 mL, some regions of the mucosa remain uncovered by the liquid film, and most regions do not yet reach their liquid-holding capacity, as demonstrated in green in Figure 4a. This is reasonable because film spreading is controlled by two competing forces: the driving force from gravity vs. the resistance from intermolecular forces (wall shear and surface tension). In many regions, the liquid film is still thin (i.e., <0.324 mm) and its weight component in the tangential wall direction is not large enough to overcome the intermolecular resistance, preventing it from spreading further, as shown in the similar film patterns between 1 s, 30 s, and 3 min through 20 min in Figure 4a. It is acknowledged that liquid evaporation and tissue absorption were not considered in this study, both of which would decrease the film height over time and reduce runoff risk.

Figure 4b shows the variation of the liquid film with time after a spray dose of 0.50 mL into the right nasal passage. With a spray dose twofold of that in Figure 4a, a larger portion of the nasal passage is covered by the liquid film. The initial distributions of the spray droplets are the same regardless of the nasal spray doses; however, gravity and surface tension redistribute the liquid film. With the subject in an inclined body position of 45°, a small portion of the liquid film extends beyond the turbinate region and enters the nasopharynx (Figure 4b). No difference is observed in the liquid distribution at 30 s, 1 min, and 3 min. This is expected because a force equilibrium is reached between the gravitational force and the surface tension, with no absorption and evaporation.

**Figure 5:**
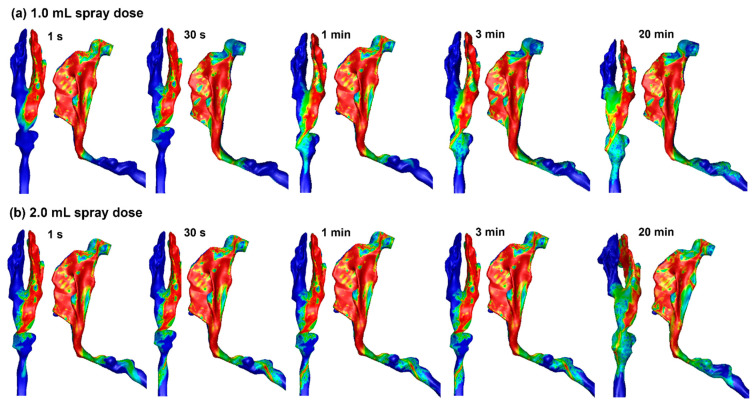
Liquid film translocation vs. time in the right nasal passage with a 45° back tilt position and a spray dose of (**a**) 1.0 mL and (**b**) 2.0 mL.

Figure 5 shows the liquid film evolution after spraying 1.0 mL and 2.0 mL doses into the right nasal passage. Clearly, the nasal mucosa cannot hold the applied dose even though nearly all nasal walls have reached their holding capacities. The liquid film moves, driven by gravity while hurdled by wall shear and surface tension, into the nasopharynx, pharynx, and even the throat. These runoffs can trigger adverse responses such as a sense of bitterness, coughing, or choking. Because both the 1.0 mL (Figure 5a) and 2.0 mL (Figure 5b) doses exceed the nasal wall holding capacity, the liquid film distributions appear similar between these two spray doses.

#### 2.4.2. Delivered Dose and Runoff in Supine Position

**Figure 6:**
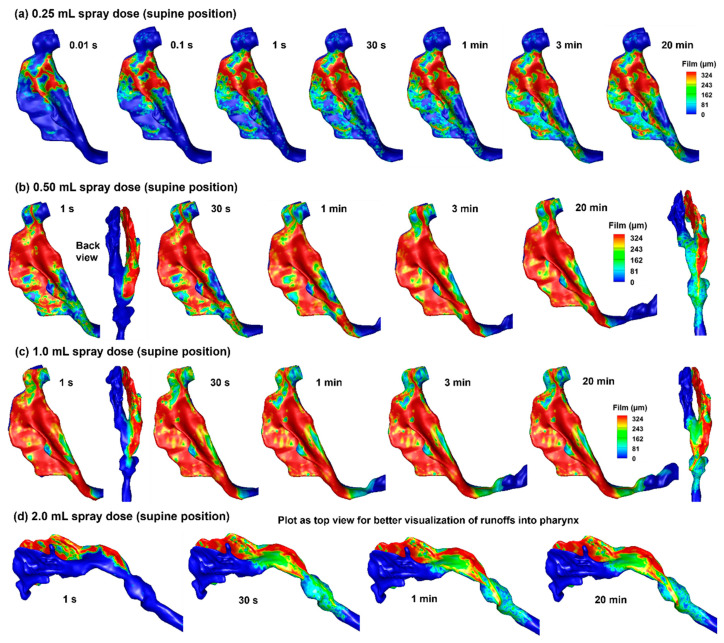
Liquid film translocation vs. time in the right nasal passage with a supine position and four doses: (**a**) 0.25 mL, (**b**) 0.50 mL, (**c**) 1.0 mL, and (**d**) 2.0 mL. Note: the 2.0 mL cases are plotted as a top view for better visualization of runoff into the pharynx.

Simulations were also conducted for spray distributions in the 10-day-old nose model in a supine position with spray doses of 0.25 mL, 0.50 mL, 1.0 mL, and 2.0 mL, as shown in Figure 6a–d. With a spray dose of 0.25 mL (Figure 6a), the liquid film stabilizes at 1 s and exhibits no significant variation after that. No runoff into the pharynx is observed in this case, even though spray droplets are noted in the nasopharynx (Figure 6a). For a spray dose of 0.50 mL (Figure 6b), the liquid film appears to stabilize at 1 s; however, subtle changes in the liquid film can also be observed after that. Significant deposition is observed in that nasopharynx (NP), with an appreciable fraction entering the pharynx and larynx (Figure 6b). The liquid film development with a spray dose of 1.0 mL appears similar to that with 0.50 mL, except that the film spreads earlier into the pharynx region (Figure 6c vs. Figure 6b).

From Figure 6d, it is clear that the infant nose (right nasal passage in this case) cannot hold the spray dose of 2.0 mL. Based on a surface area of 14.35 mm^2^ of the right nasal passage, a spray dose of 2.0 mL gives rise to an average film thickness of 1.238 mm, which is much larger than the maximum film thickness of approximately 0.324 mm. As a result, significant runoff occurs, which can reach and irritate the throat.

**Figure 7:**
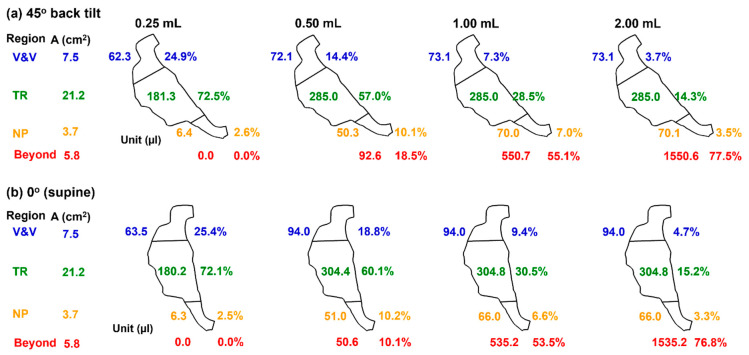
Comparison of dose distribution in the 10-day-old nose model among four spray doses (0.25 mL, 0.50 mL, 1.0 mL, and 2.0 mL) in a body position of (**a**) 45° back tilt, and (**b**) 0° (supine).

A summary of delivered doses in different anatomical regions is presented in Figure 7, including the nasal vestibule and valve (V&V), the turbinate region, the nasopharynx (NP), and beyond (pharynx and larynx). The doses in the turbinate are colored green, indicating the targets. By contrast, the doses in the NP are colored brown, and those beyond are colored red, indicating the conditions that should be avoided and must be avoided, respectively. Runoffs become apparent beginning with the spray dose of 0.50 mL. The deposition fraction (DF) beyond the NP is 18.5% in the 45° back tilt position and 10.1% in the supine position. Moreover, excess spray volumes in the 1.0 mL and 2.0 mL cases all enter the pharynx, regardless of the body position, indicating that the 0.50 mL spray dose has exceeded the liquid-holding capacity of the right passage of this infant nose model.

### 2.5. Comparison of Results Using Maximum Film Height, h_max_, of 0.324 mm vs. 0.220 mm

The liquid-holding capacity of a surface (i.e., the maximum film height, *h_max_*) varies with angle. However, the Eulerian Wall-Film (EWF) model in ANSYS Fluent 2025 can only consider a constant *h_max_* and thus cannot directly account for its dependance on wall angle. To evaluate the influence of *h_max_* on deposited/runoff doses, we compared EWF-predicted results using two choices of *h_max_*: 0.324 mm on an inclined wall of 45° vs. 0.220 mm on a vertical wall (i.e., 90°). The first was intended to represent the averaged dose, while the second was considered the worst-case scenario, assuming the majority of airway walls were vertical (90°). Two head positions were considered as follows: 45° back tilt and 0° (supine).

#### 2.5.1. 45° Back Tilt Head Position

**Figure 8:**
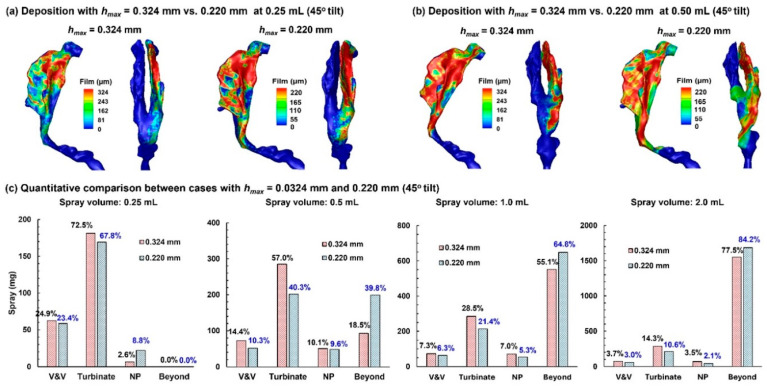
Comparison of dose distributions predicted with *h_max_* = 0.324 mm vs. *h_max_* = 0.220 mm in a 45° back tilt position: (**a**) final deposition distributions with a 0.25 mL dose, (**b**) final deposition distributions with a 0.50 mL dose, and (**c**) quantitative comparison of regional deposition for applied spray volumes of 0.25 mL, 0.50 mL, 1.0 mL, and 2.0 mL.

Figure 8a compares the predicted dose distributions with 0.324 mm *h_max_* and 0.220 mm *h*_max_ after a spray dose of 0.25 mL with a 45° inclined head position.

With a smaller wall-film holding capacity, the dose distribution predicted with 0.220 mm *h_max_* is more spread out than its 0.324 mm counterpart. All doses are deposited in the nose, with no runoff detected beyond the nasopharynx (NP), even in the worst-case scenario (0.220 mm case). The first panel in Figure 8c compares the regional deposition rates after applying 0.25 mL. As expected, more doses deposit in the vestibule–valve (V&V) and turbinate region using 0.324 mm. It is important to note that the dose in each subregion is NOT proportional to 324/220, indicating other influences on the wall-film development, such as airway geometrical details (convexity, concavity, curvatures, etc.).

Results with a spray of 0.50 mL are shown in Figure 8b. Again, the 0.220 mm case, with a smaller wall-film holding capacity, shows a greater spreading distance compared to the 0.324 mm case. Less deposition in the 0.220 mm case is observed in the V&V and turbinate region, while the dose beyond the NP doubled than the 0.324 mm case, i.e., 18.5% vs. 38.8% (second panel in Figure 8c). Considering an identical initial deposition, these differences indicate a large impact of the choice of *h_max_* on liquid dose translocation.

Further comparison of dose distributions with a 1.0 mL and 2.0 mL spray is shown in the third and fourth panels of Figure 8c, respectively. Similar to the 0.50 mL case, the predicted doses in the V&V, turbinate, and nasopharynx (NP) are lower using 0.220 mm *h_max_*, while the predicted runoff is higher. It should be noted that a spray dose of 0.50 mL already exceeds the wall-film holding capacity of the 10-day-old nose (single passage), and any larger spray dose is wasted as runoff.

#### 2.5.2. Supine Position (0°)

**Figure 9:**
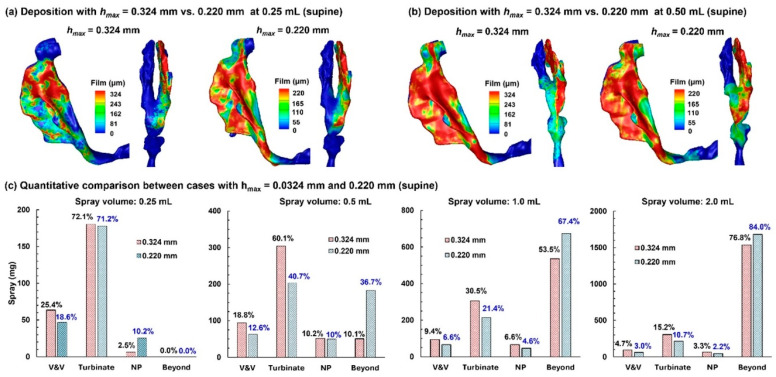
Comparison of dose distributions predicted with *h_max_* = 0.324 mm vs. *h_max_* = 0.220 mm in a supine position: (**a**) final deposition distributions with a 0.25 mL dose, (**b**) final deposition distribution with a 0.50 mL dose, and (**c**) quantitative comparison of regional deposition for applied spray volumes of 0.25 mL, 0.50 mL, 1.0 mL, and 2.0 mL.

Predicted dose distributions in the supine position using 0.324 mm *h_max_* vs. 0.220 mm *h_max_* are compared in Figure 9a and Figure 9b for the spray doses of 0.25 mL and 0.50 mL, respectively. For both options of *h_max_*, no deposition beyond the NP is observed with 0.25 mL (Figure 9a), while appreciable doses occur with 0.50 mL (Figure 9b). In both cases, liquid shifts further downward when using 0.220 mm *h_max_*, reflecting a lower wall liquid-holding capacity that allows more coverage of airway walls for a given dose.

The quantitative comparison of regional doses between predictions using 0.324 mm and 0.220 mm *h_max_* is presented in Figure 9c for spray doses of 0.25 mL, 0.5 mL, 1.0 mL, and 2.0 mL. Clearly, the retained doses in the V&V and turbinate are lower when using 0.220 mm *h_max_* than 0.324 mm *h_max_*. This leads to a much higher dose beyond the NP predicted using 0.220 mm *h_max_* (for example, 36.7% vs. 10.1% with 0.324 mm *h_max_* for the 0.5 mL spray dose, Figure 9c).

Dose distributions using 0.324 mm and 0.220 mm *h_max_* in the supine position are compared in the third and fourth panels of Figure 9c for spray doses of 1.0 mL and 2.0 mL, respectively. For both cases, lower doses are observed in the V&V, turbinate, and NP, while higher doses are observed beyond the NP, indicating that the first three regions all have reached their liquid-holding capacity, with the excess liquid overflowing into the pharynx. This is consistent with approximately the same dose in the V&V (or turbinate, or NP) between the 1.0 mL and 2.0 mL cases for a given *h_max_*. For instance, for both spray doses using 0.324 mm *h_max_*, the V&V dose is 0.094 mL, the turbinate dose is 0.0304–0.305 mL, and the NP dose is 0.066 mL. In comparison, consistently lower doses are predicted in each region using 0.220 mm *h_max_* for both spray doses (1.0 mL and 2.0 mL), with 0.060–0.066 mL in the V&V, 0.214 mL in the turbinate region, and 0.044–0.045 mL in the NP, as presented in the third and fourth panels of Figure 9c.

Table 3 summarizes the predicted runoff from the right nasal passage for four spray doses and two body positions, with the 0.324 mm-*h_max_* predictions representing averaged runoffs and the 0.220 mm-*h_max_* predictions representing the worst-case scenarios. Note that runoff here includes both deposition in the NP and beyond, based on the rationale that any dose beyond the turbinate is wasted and has the potential to cause irritation. The difference between the predictions with 0.324 mm and 0.220 mm *h_max_* were also calculated (in both volume and percentage), indicating the varying impacts (i.e., 5.3–26.4%) from the choice of *h_max_* on runoff predictions using the current Fluent EWF model, depending on the spray dose. The largest impact is observed for the 0.50 mL spray dose, regardless of the body position. The 0.50 mL spray volume is also the first case in which runoff becomes apparent among the four volumes considered hereof, suggesting the necessity of carefully choosing prescribed doses for infants.

## 3. Discussion

In this study, we measured spray parameters, tracked spray droplet deposition, and modeled post-deposition liquid translocation constrained by a wall-holding capacity informed by ex vivo mucosal measurements, thereby capturing the transition from retained intranasal liquid to runoff into the nasopharynx and pharynx. Clinically relevant spray volumes (0.25–2.0 mL) were evaluated in an anatomically accurate 10-day-old nose model at two body positions (45° inclined and supine) in a coma-like state. Predictions showed that a 0.25 mL dose was fully retained in the nose, whereas 0.50 mL produced measurable runoff (≈10–19% depending on posture); 1.0 and 2.0 mL exceeded the mucosal holding capacity and yielded substantial runoff (53–77%). These findings quantify how neonatal nasal geometry and spray volume together determine usable intranasal dose, providing data to inform emergency naloxone administration. Specific findings are discussed below.

### 3.1. Mucosa Liquid-Holding Capacity h_max_ vs. Tilt Angle

This study provides a validated dataset of naloxone liquid layer thicknesses on ex vivo porcine nasal mucosa at varying angles (Figure 1b, Table 1 and Table 2). Theoretical film thicknesses, calculated from retained liquid mass per mucosal area, closely matched five-point average thicknesses obtained by grayscale image analysis. Across 70 trials, the theoretical values exceeded measured ones by less than 4%. Several factors explain why the measured thickness values were consistently lower than the theoretical ones. Any liquid loss from the mucosa sample, whether through evaporation or tissue absorption, would reduce the measured thickness. Conversely, liquid loss from the wetted cardboard (runoff dose) decreases the runoff mass and consequently increases the calculated retention mass (i.e., applied mass minus runoff). This would raise the theoretical liquid thickness and further enlarge the gap between theoretical and measured values. As a result, the small discrepancy (less than 4%) between the two methods indicates that both approaches accurately captured the retained liquid thickness across all tested angles.

This dataset was utilized in this study as modeling bounds for runoff risk assessment in a 10-day-old nose model. At the current stage, Fluent’s Eulerian Wall-Film model cannot directly consider angle-dependent *h_max_*. In other words, the maximum liquid thickness *h_max_* is a global constraint across all anatomical surfaces without considering local wall orientations. To evaluate the influences of *h_max_* runoff predictions, two experimentally measured *h_max_* were compared as follows: using 0.324 mm (on a 45° wall) as an “average-geometry” bound and to represent an average scenario, and 0.220 mm (on a vertical wall) as a conservative worst-case scenario. For a 10-day-old nose as in this study, applying 0.25 mL caused no runoff for either *h_max_*, even though the liquid film in the 0.220 mm case spread further than the 0.324 mm case, regardless of body position (Figure 8a and Figure 9a). When administering a 0.50 mL dose, the 0.220 mm case produced roughly double the runoff compared with the 0.324 mm case (Table 3, Figure 8b and Figure 9b), reflecting the significant effects of both the liquid–wall adhesion and gravity. When the administered dose is larger than 1.0 mL, all 0.220 mm cases shifted more liquid to runoff than their 0.324 mm counterparts for both body positions (Table 3). Hence, dosing/labeling and device design should prioritize low per-actuation volumes and/or adhesion-enhancing formulations.

### 3.2. Mechanistic Interpretation

Post-deposition liquid film translocation governed the eventual distribution more than the initial droplet landing pattern: once droplets collect and coalesce on the mucosa, gravity, interfacial shear, and surface tension drive film spread toward lower and surrounding regions. To constrain film growth without a direct liquid–wall adhesion model, we used maximum film thickness (*h_max_*) values derived from ex vivo mucosa experiments (0.324 mm at 45°, and 0.220 mm at 90°), showing that a smaller *h_max_* leads to greater spread and higher runoff, particularly when the spray volume is larger than 0.50 mL. This behavior aligns with the emerging CFD literature demonstrating that liquid–wall interaction and post-deposition liquid motion are critical determinants of dose delivery and cannot be captured by a deposit-on-touch assumption [28,29].

Prior CFD and in silico studies in adults and older pediatrics have shown that posterior delivery is sensitive to device plume, droplet size/velocity, head orientation, and that liquid film translocation can materially change where the drug ends up after the initial impact [28,34]. Recent methods in papers explicitly incorporating wall-film physics corroborate our central conclusion: post-deposition motion is often the rate-limiting step for effective dosing to target regions (and a key pathway for runoff) [29]. Additionally, pediatric morphometric studies highlight that infant nasal dimensions (small fossae and narrow passages) create inherently lower liquid-holding capacity than in older children, consistent with our simulated capacity threshold [35,36,37,38]. Note that the current computational framework is anatomy-specific (10-day-old) and physiology-based (ex vivo liquid retention measurement). With appropriate modifications, the framework could be readily extended to other pediatric and adult age groups, as well as to respiratory disease by incorporating disease-related anatomical or physiological changes (e.g., airway narrowing, altered surface properties, or mucus characteristics).

### 3.3. Clinical Relevance for Intranasal Naloxone in Neonates

In emergencies where IV/IM access is delayed, intranasal naloxone is attractive; however, deliverable volume is a practical constraint for neonates [39,40]. Our results suggest 0.25 mL (per nostril, single passage modeled) is within the holding capacity for a 10-day-old nose under compromised breathing, whereas ≥ 0.50 mL incurs measurable runoff and ≥1.0 mL produces substantial loss to the pharynx, potentially provoking cough/choke responses and reducing usable intranasal dose. These findings complement neonatal guidance that emphasizes careful dosing/titration and vigilant airway management, rather than endorsing large intranasal volumes [41,42]. While clinical dosing must follow institutional protocols, our results support volume-limited, multi-puff strategies and careful head positioning to minimize runoff when IN delivery is chosen for neonates in a coma-like state [43].

The posture-dependent differences in runoffs at 0.50 mL, as shown in Figure 7, highlight the significant impact of body posture on intranasal drug delivery outcomes in pediatric populations [44]. For the 10-day-old infant model, a 0.50 mL spray produced markedly different outcomes depending on position, i.e., 18.5% runoff in the 45° inclined position vs. 10.1% in the supine position. This variation indicates that seemingly minor changes in patient orientation can substantially alter mucosal drug retention and the degree of unwanted pharyngeal exposure. Clinically, this raises concerns about inconsistent dosing in neonates, where high sensitivity to even small changes in delivered doses may lead to compromised therapeutic effect or unintended systemic absorption following swallowing of runoff [45,46].

For neonatal intranasal delivery, the results in this study argue for minimizing volume per actuation (not more than 0.25 mL per passage) to remain within the holding capacity of the nasal passage. Body/head postures influence the runoff, but they cannot compensate for capacity exceedance; dose volume is the dominant driver for spray runoff in the nose. Where volume per actuation cannot be reduced, formulation or device modifications that effectively enhance local holding (e.g., rheology, surface energy, plume angle, and exiting velocity) should be considered, as demonstrated in Figure 2 and Figure 3, to shift behavior toward the 0.324 mm outcome. Nevertheless, capacity exceedance still dominates at doses ≥ 1.0 mL.

### 3.4. Limitations

This study is subject to several assumptions that may limit the generalizability of the results, including the use of patient-specific nasal airway models, a monodisperse particle size distribution, a limited set of spray scenarios, and the omission of evaporation and absorption in the liquid film model. Variability in infant and pediatric nasal morphology and growth can substantially influence droplet transport, deposition, and film motion [44,47,48,49]. The use of a monodisperse particle size (66 µm) provides a reasonable estimate but neglects the polydisperse nature of real sprays, introducing uncertainty that future models should address [50]. Calmet et al. [48] modeled nasal spray particle size distributions spanning Dv50 = 10–150 µm and reported that nasal (anterior) deposition was similar (~80%) when Dv50 > 50 µm, which included the measured median size in this study (Dv50 ≈ 66 µm). Similarly, neglecting evaporation and absorption likely overestimates runoff, making the present results a worst-case scenario [51,52,53,54]. Finally, the Eulerian Wall-Film (EWF) model lacks orientation-dependent liquid–wall adhesion, requiring reliance on ex vivo *h_max_* values that may not fully reflect in vivo neonatal conditions of mucus, temperature, and humidity [55,56,57]. Addressing these limitations in future studies, through more anatomically diverse models, polydisperse spray simulations, and incorporation of in vivo mucosal dynamics, will be critical to improving the predictive accuracy and clinical relevance of nasal spray performance in pediatric populations. Specifically, future numerical developments should incorporate orientation-dependent adhesion models, supported by ex vivo experimental data, to improve predictive accuracy of post-deposition liquid transport.

Direct clinical validation of the numerical model was not conducted in this study. Although such validation is highly desirable, in vivo data on intranasal spray deposition and post-deposition liquid transport in neonates are scarce and ethically challenging to obtain, particularly in emergency or coma-like conditions. The present framework was therefore validated indirectly using experimentally measured spray characteristics and ex vivo porcine mucosa data to inform liquid-holding capacity and runoff behavior. Future work may focus on indirect clinical validation by adapting the model to clinical delivery configurations with available deposition data or by comparison with emerging in vivo or imaging-based datasets.

## 4. Materials and Methods

### 4.1. Image-Based 10-Day-Old Nose Model

**Figure 10:**
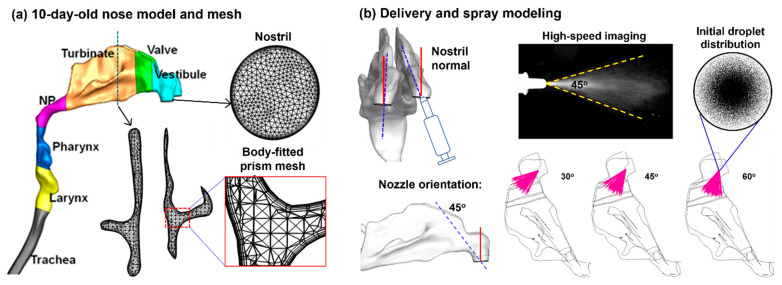
Nasal model and computational methods: (**a**) 10-day-old female nose geometry and computational mesh, and (**b**) modeling of intranasal delivery and spray administration.

An infant nasal model that was previously developed from CT scans of a 10-day-old girl (weight 3.46 kg and height 53 cm) was used in this study [38]. To quantify spray droplet deposition, the nose model was divided into seven sections: the nasal vestibule, nasal valve, turbinate region (TR), nasopharynx (NP), pharynx, and larynx (Figure 10a). The dimensions of each anatomical structure are listed in Table 4.

### 4.2. Measurement of Retained Liquid Layer Thickness vs. Tilt Angle on Porcine Nasal Mucosa

To access runoff and remaining liquid layer coverage on porcine nasal mucosa, a dose of 0.25 mL naloxone HCl formulation was sprayed onto the mucosa using a standard pre-packed syringe dispenser. The formulation was measured to have a density of 1.0088 g/cm^3^, a viscosity of 0.711 mPa.s, and a surface tension of 52.86 mN/m. The target mucosa in each case was a fresh 20 mm × 20 mm piece of suckling pig nasal mucosa, used within 4 h of slaughter to maintain freshness. The spray was focused at the center of the mucosa from a fixed tip-to-mucosa distance of 10 mm, with the tip perpendicular to the mucosa positioned vertically. The mucosa was mounted on an absorbent cardboard to collect any runoff, which was quantified by the mass increase in the board. Seven cardboard tilt angles were tested to evaluate variation in liquid-holding capacity with wall orientation, ranging from 0° to 180° in 30° increments. For each angle, ten measurements were taken for statistical significance.

Liquid film thickness measurements were based on grayscale analyses of images tangent to the surface. One minute after spraying, five high-resolution images were taken across each sample to detect and measure film thickness(es). These measurements were collected at positions 2 mm, 6 mm, 10 mm, 14 mm, and 18 mm from the lower edge (bottom edge) of the tilted samples, as shown in Figure 1. The mucosa was subsequently removed from the absorbent cardboard, which was weighed again, with the mass increase being the runoff mass for that experiment. The applied spray mass of naloxone HCL was determined by weighing the syringe before and after dispensing. Subtracting the runoff mass from the applied mass produced the nominal dose deposited on the mucosa, which was further divided by the mucosa area (4 cm^2^) and formulation density (1.0088 g/cm^3^) to estimate the theoretical liquid film thickness on the mucosa. This theoretical film thickness was compared with the average film thickness on the sample from the five optically measured values.

### 4.3. Spray Characterization and Modeling

The size distribution of the nasal sprays droplets was measured using laser diffraction (SprayLink, Dickinson, TX, USA) with 5 trials. The spray plume angle and droplet exit speeds were estimated using a particle imaging velocimetry (PIV) system. A high-speed camera captured the droplet motion at 150 frames per second for 2 s. The spray plume angle was measured to be around 45° (Figure 10b). A laser sheet (488 nm, 100 mW, OXLasers, Shanghai, China) was utilized to increase the contrast between the droplet cloud and the ambient air. The calculated droplet speed was estimated to be around 8.5 ± 3.5 m/s, which was implemented in the spray model for subsequent numerical simulations (Figure 10b).

Monodisperse aerosols (with a uniform droplet size) were generated using an in-house MATLAB (2025a) code. For the aerosols of 66 µm, a count of 1,660,770 droplets was generated to represent a single dose of 0.25 mL per actuation. The droplet count was calculated by dividing 0.25 mL by the volume of one droplet. For 0.50 mL, 1.0 mL, and 2.0 mL doses, the droplet count was scaled proportionately. The spray plume angle and the spray orientation relative to the nostril were also considered in the spray model and could be adjusted when needed (Figure 10b).

### 4.4. Computational Models

Incompressible flow and isothermal conditions were assumed in this study. The flow field was simulated using the low Reynolds number (LRN) *k-ω* model. The behavior and fate of inhaled particles were simulated with a Lagrangian tracking model with user-defined functions [58]. This fluid-particle transport model has been well tested in human respiratory airways. Steady flow and zero ambient pressure at the inlet were assumed, with vacuum pressure at the tracheal outlet. A no-slip condition and perfect particle adsorption were applied on the airway wall, which was also assumed smooth and rigid. The flow rate of a 10-day-old infant was 3.8 L/min. Considering the possible coma state of the infant during spray administration, a compromised flow rate (20%, 0.76 L/min) was used in this study. Particle sizes considered ranged from 15 µm to 145 µm with a volumetric-mean diameter VMD = 66 µm. ANSYS Fluent 19 was utilized to solve the governing mass and momentum conservation equations. Convergence sensitivity analyses were performed to establish grid-independent and particle-count-independent results. The final mesh size was 1.8 million, and the final number of test particles was 1,660,770 to match the dose of 0.25 mL drug content.

The Eulerian Wall-Film model was used to simulate liquid film translocation after initial deposition. The governing equations for the mass and momentum conservation of the wall-film are

(1) $$\frac{\partial h}{\partial t}+\nabla_{s}\cdot \left(h{\vec{V}}_{l}\right)=\frac{{\dot{m}}_{s}}{\rho_{l}}$$

(2) $$\nabla_{s}\cdot \left(h{\vec{V}}_{l}{\vec{V}}_{l}\right)=-\frac{h\nabla_{s}\left(P_{g}+P_{h}+P_{\sigma }\right)}{\rho_{l}}+{\vec{g}}_{\tau }h+\frac{3}{2\rho_{l}}{\vec{\tau }}_{fs}-\frac{3\nu_{l}}{h}\vec{V}+\frac{\dot{q}}{\rho_{l}}$$

In Equation (1), *ρ_l_* is the liquid density, *h* is the film height, $\vec{V}$*_l_* is the mean film velocity, ∇_s_ is the surface gradient operator, and $\dot{m_{s}}$ is the mass source due to droplet collection, film separation, and film stripping. In the right-hand-side of Equation (2), the first term represents the loading in the normal direction, and the second-to-fourth terms represent the loading in the tangential direction, while the last term is the momentum gain or loss due to droplet collection or separation. Specifically, *P_g_* is the air pressure, $P_{h}=-\rho h(\vec{n}\cdot \vec{g})$ is liquid-film-induced pressure normal to the film (spreading), and $P_{\sigma }=-\sigma \nabla_{s}\cdot \left(\nabla_{s}h\right)$ is the pressure caused by surface tension. The second term is gravitational effect tangential to the film, the third term is the viscous shear force at the air-film interface, and the fourth term is the viscous force at the film-wall interface. To calculate the viscous force, the film velocity is assumed to have a parabolic profile. In this study, the Eulerian Wall-Film (EWF) model does not have the option to consider the liquid–wall surface tension (even though the liquid-air surface tension can be considered). The closest approximation to this variable is the “maximum thickness” parameter in the thickness control of the EWF model, as shown below. Considering that the human nasal passage is like a convoluted, narrow channel, two maximum film thickness values were considered as follows: 0.324 mm (at a 45° tilt angle) to represent an average scenario, and 0.220 mm (at a 90° tilt angle) to represent a worst-case scenario. Simulation results with both 0.324 mm and 0.220 mm are presented, and their influence on the runoff predictions from the nose is compared.

### 4.5. Study Design and Numerical Methods

To evaluate the dose distribution and possible runoff of the sprays in a 10-day-old nose model, variables were considered as follows: (1) device-related: spray plume angle (45°), droplet sizes (19–113 µm, mean: 66 µm), droplet existing speed (5–12 m/s), and solution physical properties (1.0088 g/cm^3^, a viscosity of 0.711 mPa.s, and a surface tension of 52.86 mN/m); (2) patient-related: breathing rate (0.76 L/min, equivalent to 20% of normal condition), body position (45° back tilt and 0° supine), and the nasal mucosa liquid-holding capacity at varying angles; and (3) administration-related: nostril (right and left), and release position (at the nostril, or with a short insertion into the nostril). Variables of interest (outputs) include (1) initial droplet deposition; (2) film translocation at different times after spray administration (0–20 min); and (3) final doses in the vestibule and valve, turbinate, nasopharynx, and beyond.

Airflow fields and droplet motions were solved using ANSYS Fluent 23 (Canonsburg, PA, USA). Mesh generation was performed with ICEM CFD. Body-fitted prism elements were generated in the near-wall region, which had been shown to play a crucial role in matching CFD predictions and comparable measurements for both nanoparticles [59] and micrometer particles [60]. The grid independence study was conducted by testing six mesh sizes increasing from 0.5 million to 4.0 million, and the grid-independent deposition was achieved at 2.4 million, with the deposition rate of 66 µm deposition fraction (DF) variation being less than 1%. The airway walls were rigid and had a no-slip condition (i.e., u_wall_ = 0). Wall deposition was assumed whenever droplets touched the wall. A near-wall interpolation algorithm (NWI) was implemented, which had demonstrated improved predictions for both nano- and micro-meter aerosols [58].

## 5. Conclusions

Using an anatomically accurate nose model of a 10-day-old female, we quantified how spray volume and body/head posture determine intranasal retention in a coma-like state. Experimentally determined spray characteristics and mucosal liquid-layer thicknesses were implemented in models for droplet tracking and liquid translocation. Results show that a 0.25 mL dose was fully retained intranasally, whereas larger volumes exceeded the mucosal holding capacity and produced increasing runoffs into the nasopharynx. For both inclined and supine positions, runoff in the 10-day-old model was predicted to be 10.1–18.5% at 0.50 mL, 53–55% at 1.0 mL, and 77% at 2.0 mL, with the highest posture-dependence at 0.50 mL. A validated dataset for liquid layer thicknesses on ex vivo porcine nasal mucosa at varying angles is presented, which can be used to enhance the prediction accuracy of post-deposition liquid transport by incorporating orientation-sensitive liquid–wall adhesions.

## Figures and Tables

**Table 1:** Measured runoff and deposited liquid thickness on the porcine nasal mucosa at a 60° tilt.

#	Syringe (g)		Cardboard (g)		Film Thickness (µm) at Five Points (from Bottom)				
	Before	After	Before	After	2 mm	6 mm	10 mm	14 mm	18 mm
1	12.032	11.789	2.363	2.490	330	305	283	263	246
2	12.050	11.803	2.362	2.490	325	316	272	251	239
3	12.025	11.776	2.365	2.493	347	316	287	263	240
4	12.098	11.850	2.313	2.435	332	310	311	276	242
5	12.060	11.819	2.360	2.484	311	293	276	256	241
6	12.035	11.790	2.347	2.475	330	305	278	256	237
7	12.012	11.765	2.326	2.464	293	283	260	246	231
8	12.037	11.792	2.315	2.444	318	307	262	252	237
9	12.008	11.762	2.322	2.442	344	328	308	282	248
10	12.079	11.833	2.338	2.470	317	296	264	257	227

**Table 2:** Comparison between theoretical and five-point averaged thickness at a 60° tilt.

Test #	Appl. (g)	Runoff (g)	Deposited (g)	Theor. (µm)	5-p Measur. (µm)	Variation (%)
1	0.243	0.127	0.116	287	285	−0.7
2	0.247	0.128	0.119	295	281	−4.9
3	0.249	0.128	0.121	300	291	−3.1
4	0.248	0.122	0.126	310	294	−5.8
5	0.241	0.124	0.117	290	275	−5.0
6	0.245	0.123	0.117	290	281	−3
7	0.247	0.138	0.109	270	263	−2.8
8	0.245	0.129	0.116	287	275	−4.3
9	0.246	0.12	0.126	312	302	−3.3
10	0.246	0.132	0.116	300	291	−3.7
Average	0.246	0.128	0.118	293	282	−3.6
Std. Dev.	0.002	0.005	0.005	13	12	1.4
RSD (%)	0.8	3.9	4.2	4.4	4.3	-

**Table 3:** Summary of runoff (NP and beyond) from the right passage of a 10-day-old nose.

Body	*h_max_* (mm)	Runoff as Volume (mL)				Runoff as % of V0			
		0.25	0.50	1.0	2.0	0.25	0.50	1.0	2.0
45°	0.324	6.4 × 10^−3^	0.1429	0.6207	1.6207	2.6	28.6	62.1	81.0
	0.220	22.0 × 10^−3^	0.2469	0.7017	1.7270	8.8	49.4	70.1	86.3
	Variance	15.6 × 10^−3^	0.1040	0.0810	0.1063	6.2	20.8	8.0	5.3
0°	0.324	6.3 × 10^−3^	0.1016	0.6012	1.6012	2.5	20.3	60.1	80.1
	0.220	25.4 × 10^−3^	0.2334	0.7201	1.7253	10.2	46.7	72.0	86.2
	Variance	19.1 × 10^−3^	0.1318	0.1189	0.1241	7.7	26.4	11.9	6.1

**Table 4:** Nasal airway dimension of the 10-day-old female.

	Vestibule	Valve	TR	NP	Pharynx	Larynx	Total	R Nostril	L Nostril *
Volume (cm^3^)	0.58	0.21	1.57	0.48	0.31	0.36	3.51		
Area (cm^2^)	3.85	3.60	21.09	3.72	2.96	2.87	38.09	0.166	0.166
Diameter * (cm)	0.60	0.23	0.30	0.52	0.42	0.50	0.37	0.46	0.46

* The diameter of the vestibule to larynx is the effective diameter calculated as 4×Volume/Area, while the diameter of the right nostril and left nostril is the hydraulic diameter calculated as 4*A/perimeter.

## Data Availability

The data presented in this study are available upon request from the corresponding author. The data are not publicly available due to privacy.
